# A metabolic–inflammatory burden phenotype associated with urinary glucose in colorectal cancer

**DOI:** 10.3389/fcell.2026.1797508

**Published:** 2026-03-13

**Authors:** Ningzhe Yan, Wei Yan, Zhu Deng, Heping Wang, Qiuyan Guan, Jie Li

**Affiliations:** 1 Oncology Department, Guang’anmen Hospital, China Academy of Chinese Medical Sciences, Beijing, China; 2 Graduate School, Beijing University of Chinese Medicine, Beijing, China; 3 Shandong Provincial Hospital Affiliated to Shandong First Medical University, Shandong First Medical University, Jinan, China

**Keywords:** colorectal cancer, inflammation, metabolic stress, metabolic–inflammatory burden score, risk stratification, urinary glucose

## Abstract

**Background:**

Metabolic dysregulation and chronic inflammation are frequently observed in individuals with colorectal cancer (CRC), particularly in the context of diabetes-related conditions. Identifying simple clinical indicators that reflect these combined alterations remains of interest. Urinary glucose, routinely assessed in clinical practice, may capture transient metabolic stress, but its association with integrated metabolic–inflammatory characteristics in CRC has not been systematically evaluated.

**Methods:**

A hospital-based cross-sectional case–control analysis was conducted, including individuals with confirmed colorectal cancer (CRC) and non-CRC controls undergoing clinical evaluation during the same period. A composite Metabolic–Inflammatory Burden Score (MIBS) was constructed using urinary glucose status together with selected inflammatory and tumor markers. Multivariable logistic regression was performed with CRC status (1 = CRC, 0 = non-CRC) as the dependent variable, and model performance was assessed in the primary cohort. External validation was performed in an independent NHANES subset using a reduced model consistent with the variables available in that dataset.

**Results:**

Individuals with higher urinary glucose categories exhibited higher levels of systemic inflammation and tumor-related markers, along with altered immune cell profiles. Urinary glucose remained associated with CRC after adjustment for demographic, metabolic, inflammatory, and molecular factors. Incorporation of urinary glucose into the composite framework was associated with improved model discrimination in the primary analysis, and similar patterns were observed in the external NHANES cohort, including differences in predicted risk distributions across urinary glucose categories.

**Conclusion:**

Urinary glucose was associated with distinct metabolic–inflammatory characteristics in CRC and contributed to a composite burden framework that demonstrated consistent patterns in an independent population-based dataset.

## 1 Introduction

Colorectal cancer (CRC) continues to impose a substantial global health burden, with persistently high incidence and mortality across diverse populations (Biller and Schrag, 2021; Shin et al., 2023; Eng et al., 2022). In parallel, metabolic disorders, particularly type 2 diabetes mellitus (T2DM), have become increasingly prevalent worldwide (Młynarska et al., 2025). Accumulating population-based evidence indicates that individuals with diabetes are more likely to develop CRC and experience less favorable outcomes than those without diabetes (Pearson-Stuttard et al., 2021; Wang et al., 2024). These observations have prompted growing interest in the role of metabolic disturbances in colorectal carcinogenesis and disease progression.

Alterations in glucose metabolism, insulin signaling, and lipid homeostasis are frequently accompanied by systemic inflammatory responses. In patients with CRC, this combination of metabolic dysregulation and chronic low-grade inflammation represents a commonly observed clinical phenotype, especially among those with coexisting diabetes-related conditions (Zhao and Wu, 2024; Chen et al., 2021; Samuel et al., 2025; Li et al., 2022; Melia et al., 2022). Biomarkers reflecting inflammatory activity, such as C-reactive protein (CRP) and leukocyte-based indices, have been linked to tumor characteristics and clinical outcomes in colorectal cancer (Kim et al., 2023). In addition, tumor-associated markers, including carcinoembryonic antigen (CEA) and mismatch repair–related proteins such as PMS2, provide complementary information regarding tumor biology and molecular features (Zheng et al., 2022; Andresdottir et al., 2023). Together, these indicators capture different dimensions of the metabolic–inflammatory and tumor-related state in CRC.

Despite extensive investigation of glycemic and inflammatory markers, certain routinely available metabolic indicators have received limited attention in this context. Urinary glucose, commonly assessed in clinical practice, reflects episodic elevations in blood glucose that exceed the renal threshold and may indicate short-term metabolic stress. Its relationship with inflammatory markers and tumor-related characteristics in CRC has not been systematically evaluated. To address this gap, the present study integrated urinary glucose with selected inflammatory and molecular markers to construct a Metabolic–Inflammatory Burden Score (MIBS). This framework was applied to characterize metabolic–inflammatory profiles across urinary glucose categories in a cross-sectional population of individuals with colorectal cancer.

## 2 Materials and methods

### 2.1 Study design and population

This study employed a hospital-based cross-sectional case–control design. Participants included individuals with a confirmed diagnosis of colorectal cancer (CRC) (cases) and individuals without CRC (controls) who underwent clinical evaluation during the same time period. CRC status was determined based on documented clinical and pathological diagnoses.

**FIGURE 1:**
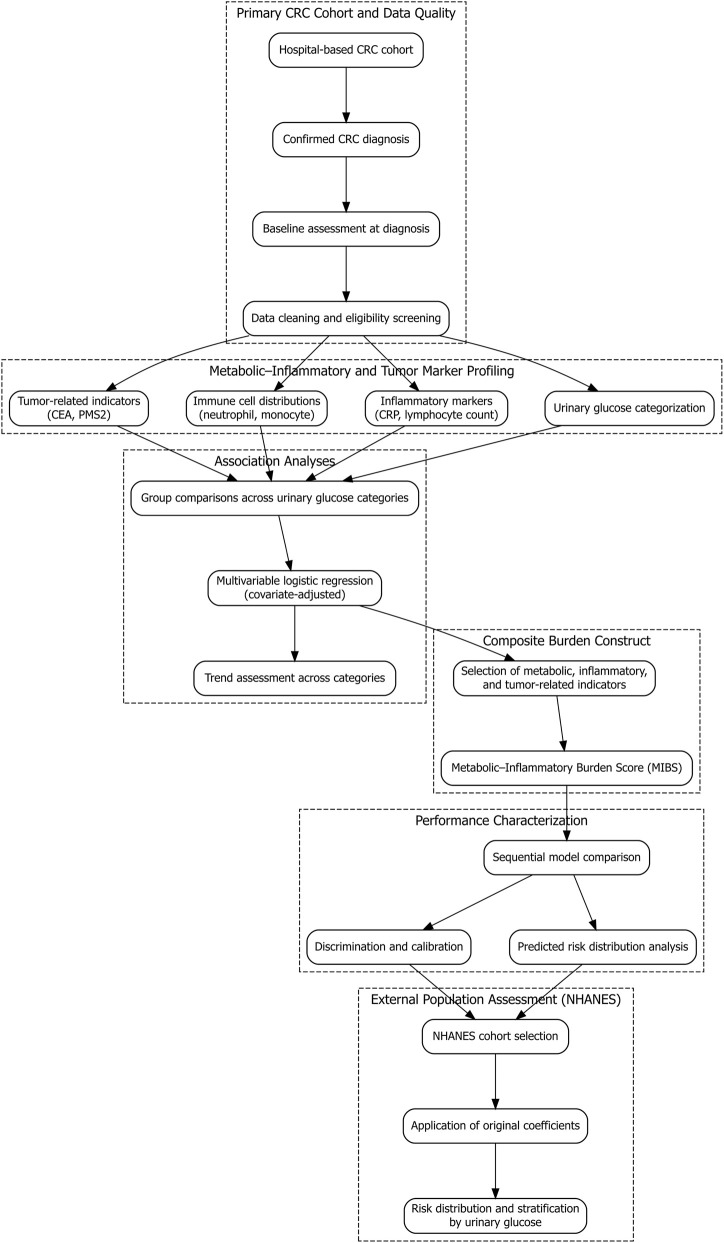
Study flowchart. Flow diagram showing participant inclusion and exclusion criteria. After screening, 1,586 individuals were included and stratified into four groups based on urinary glucose status (negative, normal, trace, positive).

Clinical assessments and laboratory measurements were performed as part of routine diagnostic procedures. Records were reviewed to ensure completeness and consistency of demographic information, laboratory measurements, and diagnostic documentation. Individuals with missing demographic variables, incomplete laboratory test results, or ambiguous diagnostic records were excluded during the data screening process. Participant selection and data processing procedures are illustrated in Figure 1.

After application of eligibility criteria and data quality checks, a total of 1,586 participants were retained for the final analytic dataset, including 378 CRC cases and 1,208 non-CRC controls. All analyses were conducted using baseline data corresponding to the diagnostic evaluation period.

### 2.2 Exposure and outcome assessment

Urinary glucose was measured as part of routine urinalysis in the hospital clinical laboratory at the time of baseline evaluation. Testing was performed using automated urine analyzers with standard dipstick strips based on the glucose oxidase method, following routine laboratory procedures and quality-control protocols.

Results were recorded according to the semi-quantitative grading system routinely used in clinical practice. Urinary glucose was categorized as negative (−), trace (±), or positive grades (1+ and above), reflecting increasing concentrations based on the dipstick colorimetric scale. In general clinical interpretation, negative indicates no detectable urinary glucose, trace reflects low-level detectable glucose, and positive grades represent progressively higher concentrations.

For the present analysis, urinary glucose results were grouped into four categories (negative, normal, trace, and positive) according to the original laboratory reporting format. These categories were treated as ordinal exposure variables in subsequent analyses, with the negative group serving as the reference category.

The outcome variable was colorectal cancer (CRC) status. CRC cases were defined based on documented clinical and pathological diagnoses in the hospital records. Individuals without a diagnosis of CRC during the same evaluation period were classified as non-CRC controls. CRC status was coded as a binary variable (1 = CRC, 0 = non-CRC) for use in multivariable logistic regression analyses.

To characterize the combined metabolic–inflammatory profile, a Metabolic–Inflammatory Burden Score (MIBS) was constructed based on urinary glucose together with selected inflammatory and tumor-related markers, including C-reactive protein (CRP), lymphocyte count, carcinoembryonic antigen (CEA), and PMS2 expression. The final MIBS value was defined as the linear predictor (logit) derived from the fully adjusted regression model.

### 2.3 Covariates

Covariates considered in the analyses included age, sex, and ethnicity. Lifestyle factors were smoking status and alcohol consumption. Marital status was classified as married, divorced, single, or widowed. Comorbidities assessed included hypertension, hyperlipidemia, and a history of colorectal polyps.

### 2.4 Statistical analysis

Multivariable logistic regression was performed with CRC status (1 = CRC, 0 = non-CRC) as the dependent variable. Urinary glucose categories were entered as categorical predictors, with the negative urinary glucose group serving as the reference category. Odds ratios (ORs) with 95% confidence intervals (CIs) were calculated to estimate associations between urinary glucose and CRC after adjustment for covariates.

Normality of continuous variables was assessed using the Shapiro–Wilk test. As several laboratory variables demonstrated non-normal distributions, group comparisons were conducted using non-parametric methods (Kruskal–Wallis test) where appropriate. Continuous variables are presented as mean ± standard deviation (SD) for descriptive consistency.

A series of four sequential models was constructed to assess the incremental contribution of different variable groups. The first model included demographic and lifestyle characteristics (age, sex, smoking, and alcohol use). The second model added inflammatory indicators (CRP and lymphocyte count). Tumor-related markers (CEA and PMS2) were incorporated in the third model. Urinary glucose category was introduced in the final model, representing the complete Metabolic–Inflammatory Burden Score (MIBS) framework. The MIBS was defined as the linear predictor (logit value) derived from the full model.

Although urinary glucose categories were unevenly distributed, multivariable logistic regression is generally robust to group size imbalance when the number of outcome events is sufficient. The trace and positive groups maintained adequate case numbers to ensure stable coefficient estimation.

### 2.5 Software

All analyses were conducted in R (version 4.3.2; R Foundation for Statistical Computing, Vienna, Austria). Data management and descriptive analyses were performed using the *tidyverse* and *tableone* packages. Group comparisons used functions from *stats* and *rstatix*. Logistic regression was implemented using *stats* and *rms*. ROC curves were created with *pROC*, calibration curves with *rms*, and DCA with the *rmda* package. Figures were produced using *ggplot2* and *cowplot*.

### 2.6 External validation using NHANES

External validation was conducted using data from the National Health and Nutrition Examination Survey (NHANES) 2015–2016 and 2017–2018 cycles. NHANES is a nationally representative, population-based survey conducted in the United States using standardized interviews, physical examinations, and laboratory assessments with a multistage probability sampling design.

Participants aged ≥40 years were considered eligible for validation in order to reflect the age distribution of colorectal cancer (CRC) risk. Data from the two survey cycles were merged according to NHANES analytic guidelines. The following variables were extracted: age (RIDAGEYR), sex (RIAGENDR), race/ethnicity (RIDRETH1), smoking status (SMQ020), high-sensitivity C-reactive protein (LBXHSCRP), lymphocyte percentage (LBXLYPCT), neutrophil percentage (LBXNEPCT), monocyte percentage (LBXMOPCT), and urinary glucose (URXUAS3).

CRC status in NHANES was defined based on self-reported physician diagnosis of colon cancer or rectal cancer. Participants were classified as having CRC (CRC = 1) if cancer-type codes corresponding to colon or rectal cancer were reported in the cancer questionnaire module, and as non-CRC (CRC = 0) if no history of these malignancies was reported. Individuals with missing cancer history data were excluded.

Because several tumor-related variables included in the primary hospital-based model (such as carcinoembryonic antigen and PMS2 expression) were not available in NHANES, external validation was conducted using a reduced multivariable model restricted to variables available in the survey dataset. Participants with missing data on urinary glucose, CRP, lymphocyte percentage, smoking status, or CRC status were excluded using a complete-case approach.

After application of eligibility criteria and exclusion of missing data, 2,246 participants were included in the validation analysis, among whom 59 reported a history of colorectal cancer.

Regression coefficients derived from the corresponding reduced model in the primary cohort were applied directly to the NHANES dataset without refitting. Predicted probabilities of CRC were calculated using the linear predictor from the primary model. Model discrimination was evaluated using receiver operating characteristic (ROC) curves and the area under the curve (AUC).

## 3 Results

### 3.1 Baseline characteristics

A total of 1,586 individuals were included in the analysis: 1,164 in the negative urinary glucose group, 211 in the normal group, 91 in the trace group, and 120 in the positive group. Baseline characteristics are summarized in Table 1. Compared with the negative group, the trace and positive groups had higher proportions of divorced individuals and current smokers, as well as a greater prevalence of colorectal polyps and hyperlipidemia. Laboratory results showed higher CRP and CEA concentrations and lower lymphocyte counts with increasing urinary glucose categories.

**TABLE 1:** Baseline characteristics of study participants by urinary glucose group.

Level	Negative	Normal	Trace (±)	Positive	p
n	1164	211	91	120	​
Sex					
Female	585 (50.3)	105 (49.8)	44 (48.4)	50 (41.7)	0.353
Male	579 (49.7)	106 (50.2)	47 (51.6)	70 (58.3)	​
Ethnicity					
Han	1048 (90.0)	185 (87.7)	83 (91.2)	107 (89.2)	0.72
Minority	116 (10.0)	26 (12.3)	8 (8.8)	13 (10.8)	​
Marital status					
Divorced	95 (8.2)	25 (11.8)	13 (14.3)	40 (33.3)	<0.001
Married	809 (69.5)	146 (69.2)	67 (73.6)	56 (46.7)	​
Single	124 (10.7)	19 (9.0)	3 (3.3)	13 (10.8)	​
Widowed	136 (11.7)	21 (10.0)	8 (8.8)	11 (9.2)	​
Smoking					
No	818 (70.3)	150 (71.1)	49 (53.8)	69 (57.5)	<0.001
Yes	346 (29.7)	61 (28.9)	42 (46.2)	51 (42.5)	​
Alcohol drinking					
No	750 (64.4)	129 (61.1)	58 (63.7)	73 (60.8)	0.73
Yes	414 (35.6)	82 (38.9)	33 (36.3)	47 (39.2)	​
Hypertension					
No	745 (64.0)	136 (64.5)	56 (61.5)	88 (73.3)	0.207
Yes	419 (36.0)	75 (35.5)	35 (38.5)	32 (26.7)	
Hyperlipidemia					
No	873 (75.0)	125 (59.2)	70 (76.9)	84 (70.0)	<0.001
Yes	291 (25.0)	86 (40.8)	21 (23.1)	36 (30.0)	​
Colorectal polyp					
No	967 (83.1)	179 (84.8)	71 (78.0)	82 (68.3)	<0.001
Yes	197 (16.9)	32 (15.2)	20 (22.0)	38 (31.7)	​
Ki-67					
High	463 (39.8)	72 (34.1)	36 (39.6)	43 (35.8)	0.411
Low	701 (60.2)	139 (65.9)	55 (60.4)	77 (64.2)	​
PMS2					
Intact	984 (84.5)	177 (83.9)	78 (85.7)	99 (82.5)	0.918
Loss	180 (15.5)	34 (16.1)	13 (14.3)	21 (17.5)	​
Age, years (mean ± SD)	61.50 (8.08)	62.20 (7.52)	62.65 (8.51)	62.06 (7.45)	0.377
Hemoglobin, g/L (mean ± SD)	136.75 (16.24)	137.16 (15.81)	135.97 (18.30)	138.72 (15.19)	0.583
Red blood cell count, ×10^12^/L (mean ± SD)	4.51 (0.48)	4.51 (0.49)	4.46 (0.49)	4.50 (0.52)	0.798
C-reactive protein, mg/L (mean ± SD)	2.47 (1.01)	2.83 (1.17)	3.44 (1.29)	4.14 (1.47)	<0.001
Lymphocyte count, ×10^9^/L (mean ± SD)	2.01 (0.40)	1.89 (0.37)	1.65 (0.31)	1.61 (0.31)	<0.001
Neutrophil percentage, % (mean ± SD)	60.50 (10.17)	60.67 (10.56)	62.31 (9.22)	59.56 (9.78)	0.269
Monocyte percentage, % (mean ± SD)	7.13 (1.94)	6.98 (1.99)	7.49 (1.80)	7.11 (2.14)	0.239
Carcinoembryonic antigen, ng/mL (mean ± SD)	4.39 (2.07)	5.47 (2.64)	5.95 (2.39)	7.07 (2.86)	<0.001

Data are presented as mean ± standard deviation for continuous variables and number (percentage) for categorical variables. Significant differences were observed for marital status, smoking, hyperlipidemia, colorectal polyps, CRP, lymphocyte count, and CEA, levels across groups.

### 3.2 Inflammatory markers

**FIGURE 2:**
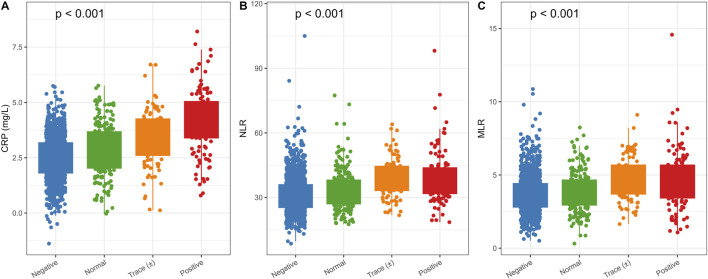
Distribution of inflammatory markers across urinary glucose categories. **(A)** CRP levels increased significantly from negative to positive groups (Kruskal–Wallis, p < 0.001). **(B)** NLR values rose progressively across urinary glucose categories (p < 0.001). **(C)** MLR levels showed a similar upward trend (p < 0.001).

Figure 2 presents the distribution of inflammatory indicators across urinary glucose groups. CRP values differed significantly across categories (Kruskal–Wallis test, p < 0.001). The neutrophil-to-lymphocyte ratio (NLR) also varied across groups (p < 0.001), with the highest values observed in the positive urinary glucose category. A similar pattern was observed for the monocyte-to-lymphocyte ratio (MLR) (p < 0.001).

### 3.3 Molecular markers

**FIGURE 3:**
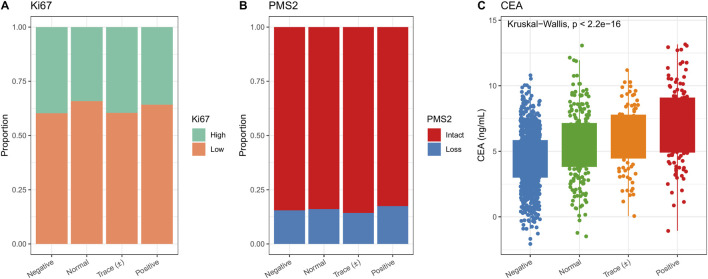
Distribution of molecular markers across urinary glucose categories. **(A)** Proportion of patients with high Ki-67 expression did not differ among groups. **(B)** PMS2 loss was more frequent in the positive urinary glucose group. **(C)** CEA levels increased significantly across groups, with the highest values in the positive group (p < 0.001).

Distributions of molecular markers are shown in Figure 3. The proportion of individuals with high Ki-67 expression was comparable across urinary glucose groups. PMS2 loss occurred more often in the positive category. CEA levels increased across categories, with the highest values in the positive group (p < 0.001).

### 3.4 Multivariable analysis

**FIGURE 4:**
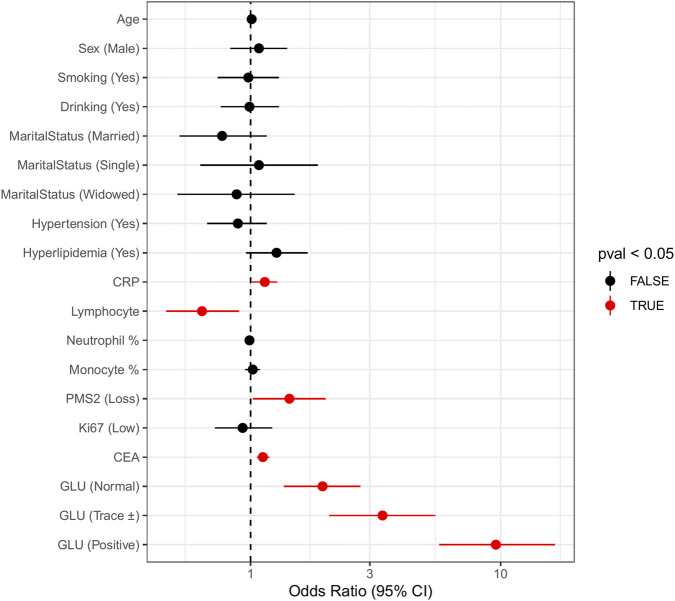
Multivariable logistic regression analysis of CRC status (CRC vs. non-CRC). Forest plot showing odds ratios (ORs) and 95% confidence intervals (CIs) for demographic, lifestyle, metabolic, inflammatory, and molecular variables. Significant predictors included CRP, lymphocyte count, CEA, PMS2 loss, and urinary glucose categories.

Estimates from the multivariable logistic regression model predicting CRC status are presented in Table 2 and Figure 4. Higher CRP (OR = 1.14, 95% CI: 1.01–1.28, p = 0.028), lower lymphocyte counts (OR = 0.64, 95% CI: 0.46–0.90, p = 0.010), higher CEA levels (OR = 1.12, 95% CI: 1.06–1.19, p < 0.001), and PMS2 loss (OR = 1.43, 95% CI: 1.02–2.00, p = 0.038) were independently associated with CRC.

**TABLE 2:** Multivariable logistic regression analysis of predictors for colorectal cancer.

Variable	OR (95% CI)	p value
Age, years	1.01 (0.99–1.02)	0.511
Sex (Male vs. Female)	1.08 (0.83–1.40)	0.555
Smoking (Yes vs. No)	0.98 (0.74–1.30)	0.898
Alcohol drinking (Yes vs. No)	0.99 (0.76–1.30)	0.968
Marital status		
Married vs. Divorced	0.77 (0.52–1.16)	0.206
Single vs. Divorced	1.08 (0.63–1.86)	0.77
Widowed vs. Divorced	0.88 (0.51–1.50)	0.634
Hypertension (Yes vs. No)	0.89 (0.67–1.16)	0.394
Hyperlipidemia (Yes vs. No)	1.27 (0.96–1.69)	0.097
C-reactive protein, mg/L	1.14 (1.01–1.28)	0.028*
Lymphocyte count, ×10^9/L	0.64 (0.46–0.90)	0.010*
Neutrophil percentage, %	0.99 (0.98–1.01)	0.321
Monocyte percentage, %	1.02 (0.95–1.09)	0.589
PMS2 (Loss vs. Intact)	1.43 (1.02–2.00)	0.038*
Ki-67 (Low vs. High)	0.93 (0.72–1.22)	0.606
Carcinoembryonic antigen, ng/mL	1.12 (1.06–1.19)	<0.001***
Urinary glucose		
Normal vs. Negative	1.94 (1.36–2.75)	<0.001***
Trace (±) vs. Negative	3.37 (2.06–5.48)	<0.001***
Positive vs. Negative	9.57 (5.68–16.52)	<0.001***

Odds ratios (ORs) with 95% confidence intervals (CIs) are shown. Independent predictors included CRP (OR, 1.14, p = 0.028), lymphocyte count (OR, 0.64, p = 0.010), CEA (OR, 1.12, p < 0.001), PMS2 loss (OR, 1.43, p = 0.038), and urinary glucose categories (normal, trace, positive vs. negative, all p < 0.001).

Neutrophil and monocyte percentages were not statistically significant but remained in the subsequent composite-score analysis because of their observed trends.

Urinary glucose categories showed graded increases in odds relative to the negative group: normal (OR = 1.94, 95% CI: 1.36–2.75), trace (OR = 3.37, 95% CI: 2.06–5.48), and positive (OR = 9.57, 95% CI: 5.68–16.52) (all p < 0.001). Variables from this model contributed to the development of the composite Metabolic–Inflammatory Burden Score (MIBS).

### 3.5 Model performance

**FIGURE 5:**
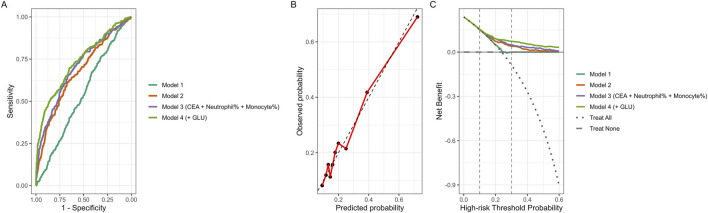
Predictive performance of sequential models. **(A)** Receiver operating characteristic (ROC) curves illustrating model discrimination. **(B)** Calibration plot of the full model (Model 4). Black points represent observed event rates within deciles of predicted probability. The red line connects observed probabilities across deciles, and the dashed gray line indicates the ideal 45° reference line representing perfect calibration. **(C)** Decision curve analysis (DCA) depicting net clinical benefit across different threshold probabilities. Model 1 included demographic and lifestyle factors (Age, Sex, Smoking, Drinking); Model 2 added inflammatory markers (CRP and Lymphocyte count); Model 3 further incorporated tumor and inflammatory cell markers (CEA, Neutrophil percentage, and Monocyte percentage); and Model 4 additionally included urinary glucose (MIBS model). The full MIBS model demonstrated the highest discrimination and net clinical benefit within the clinically relevant threshold range (0.1–0.3).

Model performance is detailed in Table 3 and Figure 5. The model including only demographic and lifestyle characteristics yielded an AUC of 0.54 (95% CI: 0.50–0.57). Adding CRP and lymphocyte count increased the AUC to 0.67 (95% CI: 0.64–0.70). Incorporating CEA and inflammatory cell percentages increased discrimination to 0.70 (95% CI: 0.67–0.73). The model that additionally included urinary glucose (MIBS) reached an AUC of 0.73 (95% CI: 0.70–0.76). Calibration curves suggested reasonable agreement between estimated and observed values, and decision curve analysis indicated higher net benefit for the MIBS model within threshold probabilities commonly used for clinical stratification.

**TABLE 3:** Discrimination performance of sequential predictive models.

Model	Variables	AUC (95% CI)	*p*-value (vs. previous model)
Model 1	Age, Sex, Smoking, Drinking	0.54 (0.50–0.57)	Reference
Model 2	Model 1 + CRP, Lymphocyte	0.67 (0.64–0.70)	4.5 × 10^−10^
Model 3 (CEA + Neutrophil% + Monocyte%)	Model 2 + CEA, Neutrophil%, Monocyte%	0.70 (0.67–0.73)	0.00216
Model 4 (+GLU)	Model 3 + Urinary glucose (MIBS)	0.73 (0.70–0.76)	0.000333

Model 1 included demographic and lifestyle factors. Model 2 added inflammatory markers (CRP, and lymphocyte count). Model 3 further incorporated tumor and inflammatory cell markers (CEA, neutrophil percentage, and monocyte percentage). Model 4 additionally included urinary glucose (MIBS, model). The AUC, increased stepwise from 0.54 in Model 1 to 0.73 in Model 4, indicating progressively improved predictive accuracy and clinical applicability.

### 3.6 External validation in NHANES

External validation was conducted in an independent subset of the National Health and Nutrition Examination Survey (NHANES) 2015–2018 cycles. After restricting to participants aged ≥40 years and applying complete-case criteria, 2,246 individuals were included in the validation analysis, among whom 59 reported a history of colorectal cancer.

**FIGURE 6:**
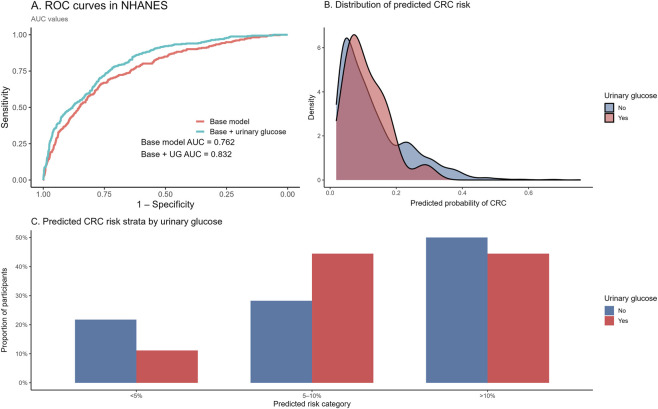
External validation of the metabolic–inflammatory burden framework in the NHANES cohort. **(A)** Receiver operating characteristic (ROC) curves comparing the base model and the model incorporating urinary glucose in the NHANES dataset. The area under the curve (AUC) is shown for each model. **(B)** Distribution of model-estimated colorectal cancer (CRC) probabilities stratified by urinary glucose status. Density curves represent the distribution of predicted probabilities for participants with and without urinary glucose. **(C)** Proportions of participants across predefined predicted risk categories (<5%, 5%–10%, and >10%) according to urinary glucose status in NHANES. Predicted risk categories were derived from the same model applied in the primary analysis.

Regression coefficients derived from the corresponding reduced multivariable model in the primary cohort were applied directly to the NHANES dataset. Receiver operating characteristic (ROC) curves are presented in Figure 6A. In the NHANES subset, the base model yielded an area under the curve (AUC) of 0.762, while the model additionally incorporating urinary glucose achieved an AUC of 0.832.

Figure 6B displays the distribution of predicted colorectal cancer (CRC) risk probabilities stratified by urinary glucose status. Predicted probabilities for both groups were predominantly concentrated at lower values. Participants with urinary glucose positivity exhibited a rightward shift in the distribution of predicted risk relative to those without urinary glucose, with higher density observed in intermediate probability ranges.

Participants were further classified into three predefined predicted risk categories (<5%, 5%–10%, and >10%) based on model-estimated probabilities (Figure 6C). Among participants without urinary glucose, 21.8% were categorized as <5% risk, 28.2% as 5%–10% risk, and 50.0% as >10% risk. Among participants with urinary glucose, corresponding proportions were 11.1%, 44.4%, and 44.5%, respectively. Differences in the distribution of predicted risk categories were observed between urinary glucose groups.

## 4 Discussion

This study examined metabolic–inflammatory characteristics across urinary glucose categories in individuals with colorectal cancer and assessed whether combining urinary glucose with inflammatory and tumor-related markers improved model discrimination. In multivariable analysis, several markers demonstrated quantifiable associations with CRC. Each 1 mg/L increase in CRP was associated with a 14% higher odds of CRC, whereas each 1 × 10^9^/L increase in lymphocyte count corresponded to a 36% reduction in odds. CEA levels showed a positive association, with each 1 ng/mL increment linked to a 12% increase in CRC odds, and PMS2 loss was associated with a 43% higher odds compared with intact expression.

Most notably, urinary glucose exhibited a clear graded relationship with CRC. Compared with individuals without detectable urinary glucose, those in the normal category had nearly a two-fold higher odds of CRC, those in the trace category had more than a three-fold higher odds, and those in the positive category demonstrated nearly a ten-fold higher odds. In parallel, higher urinary glucose levels were accompanied by higher CRP, altered leukocyte distributions, and increased CEA. Although the overall improvement in discrimination was modest, this gradient suggests that glucosuria may reflect broader metabolic–inflammatory profiles within this clinical population.

Epidemiological evidence has consistently reported higher colorectal cancer incidence and mortality among individuals with type 2 diabetes (Lawler et al., 2023). Studies have also described associations between fasting glucose, insulin resistance, and glucose variability with colorectal cancer risk and progression (Jun et al., 2022; Ruan et al., 2022). Research examining hyperglycemia in relation to oxidative stress, insulin-related pathways, and other metabolic alterations has been reported in diverse populations (González et al., 2023; Kasprzak, 2021; Edgar et al., 2021). Likewise, inflammatory markers such as CRP, lymphocyte-based indices, and monocyte-related measures have been associated with colorectal cancer characteristics and prognosis in multiple cohorts (Yang et al., 2023; Zhao et al., 2023; Koper-Lenkiewicz et al., 2021). Alterations in immune cell distributions and higher CEA concentrations have also been described in relation to colorectal cancer stage and disease behavior (Conroy et al., 2021; Bajwa-Ten Broeke et al., 2021; Mousavi et al., 2021; Bryant et al., 2023). In this context, the present analysis reflects how urinary glucose corresponds with these routinely used markers within a cross-sectional clinical setting.

The aim of this work was descriptive: to determine whether urinary glucose aligns with metabolic–inflammatory profiles and whether its inclusion contributes additional information when summarizing these features. Urinary glucose is widely available in routine practice and may indicate short-term glycemic burden. Its inclusion resulted in a modest improvement in discrimination, suggesting that glucosuria may capture aspects of the metabolic–inflammatory state not fully represented by the other markers included in the models.

Several limitations should be acknowledged. The cross-sectional design precludes assessment of temporal relationships, and measurements were obtained after CRC diagnosis. The distribution of urinary glucose categories was uneven, particularly in the trace group; however, regression estimates remained stable with relatively narrow confidence intervals. Residual confounding from unmeasured factors, including medication use and prior metabolic history, cannot be excluded. Long-term glycemic indicators such as HbA1c were unavailable. As this was a hospital-based cohort, generalizability to broader populations may be limited. Prospective studies are needed to confirm these findings.

## 5 Conclusion

This cross-sectional analysis examined metabolic–inflammatory characteristics across urinary glucose categories in individuals with colorectal cancer and evaluated whether urinary glucose contributes additional information when combined with inflammatory and tumor-related markers. Integrating urinary glucose into the composite model resulted in a modest improvement in discrimination compared with models based on demographic, lifestyle, and laboratory indicators alone. Urinary glucose corresponded with distinct metabolic–inflammatory profiles, suggesting that glucosuria may reflect broader metabolic and inflammatory patterns within this clinical population.

## Data Availability

The raw data supporting the conclusions of this article will be made available by the authors, without undue reservation.
